# Interpretation of Computed Tomography of the Head: Emergency Physicians versus Radiologists

**DOI:** 10.5812/traumamon.12023

**Published:** 2013-08-14

**Authors:** Ali Arhami Dolatabadi, Alireza Baratloo, Alaleh Rouhipour, Ali Abdalvand, Hamidreza Hatamabadi, Mohammadmehdi Forouzanfar, Majid Shojaee, Behrooz Hashemi

**Affiliations:** 1 Department of Emergency Medicine, Imam Hosein Hospital, Shahid Beheshti University of Medical Sciences, Tehran, IR Iran; 2 Department of Emergency Medicine, Shohada Tajrish Hospital, Shahid Beheshti University of Medical Sciences, Tehran, IR Iran; 3 Department of Pediatrics, Valiasr Hospital, Ghazvin University of Medical Sciences, Abyek, IR Iran; 4 Department of Family Medicine, University of Alberta, Edmonton, Canada

**Keywords:** Tomography, X-Ray Computed, Brain, Emergencies, Radiologist, Interpretation

## Abstract

**Background:**

Many patients are brought to crowded emergency departments (ED) of hospitals every day for evaluation of head injuries, headaches, neurologic deficits etc. CT scan of the head is the most common diagnostic measure used to search for pathologies. In many EDs the initial interpretation of images are performed by emergency physicians (EP). Since most decisions are made based on the initial interpretation of the images by emergency physicians and not the radiologists, it is necessary to assess the accuracy of interpretations made by the former group.

**Objectives:**

The objective of this study was to compare the findings reported in the interpretation of head CTs by emergency physicians and compare to radiologists (the gold standard).

**Materials and Methods:**

This was a prospective cross sectional study conducted from March to May 2009 in a teaching hospital in Tehran, Iran. All non-contrast head CTs obtained during the study period were copied on DVDs and sent separately to a radiologist, 6 emergency medicine (EM) attending physicians and 14 senior EM residents for interpretation. Clinical information pertaining to each patient was also sent with each CT. The radiologist’s interpretation was considered as the gold standard and reference for comparison. Data from EM physicians and residents were compared with the reference as well as with each other and statistical analysis was performed using SPSS 18.5.

**Results:**

Out of 544 CT scans, EM physicians had 35 false negatives and 53 false positives compared with radiologist’s interpretations (P < 0.0001). EM residents had 74 false negatives and 12 false positives compared with radiologist’s interpretations (P < 0.0001).

**Conclusions:**

Both EPs and ER residents either missed or falsely called a significant number of pathologies in their interpretations. The interpretations of EPs and ER residents were more sensitive and more specific, respectively. These findings revealed the need for increased training time in head CT reading for residents and the necessity of attending continuing medical education workshops for emergency physicians.

## 1. Background

Emergency departments (ED) are crucial entry points to healthcare services and usually overcrowded. Furthermore, the urgent nature of the medical conditions that bring patients to ED, add to the value of accurate and fast diagnosis and management (1).In recent years, the CT scan has become the diagnostic modality of choice for a host of pathophysiologies and much more readily available even in smaller centers with no on-site radiologists. Head CT scan study is one of the most common investigations which usually need to be interpreted by emergency doctors and management plans are initiated before the formal radiologist’s interpretation becomes available (2, 3). While accuracy of interpretation of brain CT scan by emergency physicians is of crucial importance, many EM residency programs do not allocate enough time to brain CT scan interpretation training (3, 4).

## 2. Objectives

The present study was designed to assess the accuracy of brain CT interpretations made by EP and EM residents.

## 3. Materials and Methods

This study was conducted in an academic teaching medical center in Tehran, Iran. Between March and May 2009, 544 patients with head CTs as part of their work up, were registered in this study. The hospital’s ethics committee approved the study. Informed consent forms were filled out by patients, their alternate decision makers or guardians in case of children. All of the head CT scans in the study period were included. The study process was initiated by assigning a code to each patient. Three identical forms with demographic information, history and physical exam of the patients were generated. A checklist for CT findings was also added to each form. Every head CT scan study was interpreted by an emergency physician and a senior emergency medicine resident, who had finished a one-month rotation in the diagnostic imaging unit. To have a reference for comparison, the 3rd copy of the CT scan was sent for interpretation by a radiologist as the diagnostic gold standard. All interpretations were done in a quiet room outside the emergency department with as much time as the interpreter needed. The interpretations provided by the emergency doctors and emergency medicine residents were compared with the radiologist’s interpretation. After compiling the data, statistical analyses were performed using SPSS 18.5 (SPSS, Chicago, Illinois) to calculate false positive, false negative, sensitivity, specificity, positive predictive value (PPV), negative predictive value (NPV), positive likelihood ratio (PLR) and negative likelihood ratio (NLR) for each group (EP and EM residents). The Chi-square and t-test was used for comparison between the groups.

## 4. Results

Head CT scans from 544 patients were enrolled in this study. Based on the reports from the radiologist, abnormalities were found in 259 (47.6%) of cases (acute). Head trauma was the most common reason for CT scan (291, 53.49%) and 23% of the patients with head traumas had no other complaints. Indications for brain CT of 253 non-trauma patients can be found in Table 1.

**Table 1.** CT Indications in Non-Traumatic Patients

CT Indications	No. (%)
**Disorientation**	5 (1.98)
**Paraparesia**	3 (1.18)
**Seizure**	22 (8.69)
**Hemiparesia**	28 (11.07)
**Hemiplegia**	4 (1.58)
**Syncope**	13 (5.14)
**Weakness**	9 (3.56)
**Loss of consciousness**	89 (35.18)
**Headache**	57 (22.53)
**Vertigo**	23 (9.09)
**Total**	253 (100)

**Figure 1.**
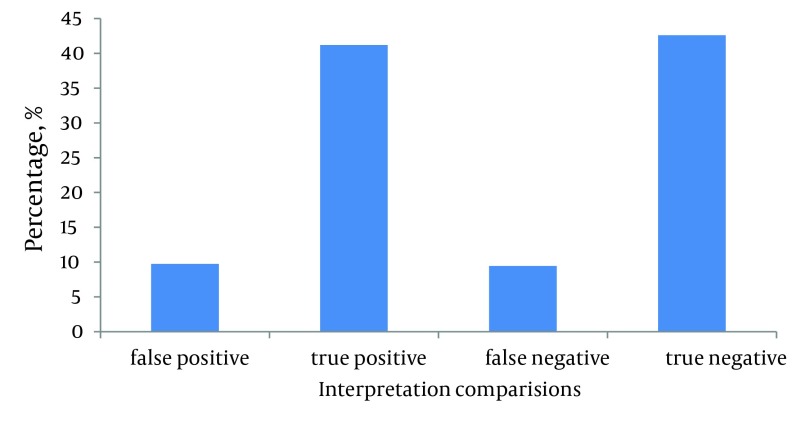
EM Attending Faculty Interpretation Correctness Percentage

The comparison between the reports from EP and radiologist, as the gold standard, revealed that 88 (16.2%, 95% CI) of the interpretations differed. There were 35 (6.44%, 95% CI) false negative and 53 (9.73%, 95% CI) false positive cases. This difference was statistically significant (P < 0.0001). These findings revealed a sensitivity and specificity of 86.5 % and 81.4% respectively for the interpretations done by the EPs. PPV of 86.9%, NPV of 86.9%, PLR of 4.6 and NLR of 0.16 were the other statistical characteristics of the interpretations of EPs (Figure 1).

**Figure 2.**
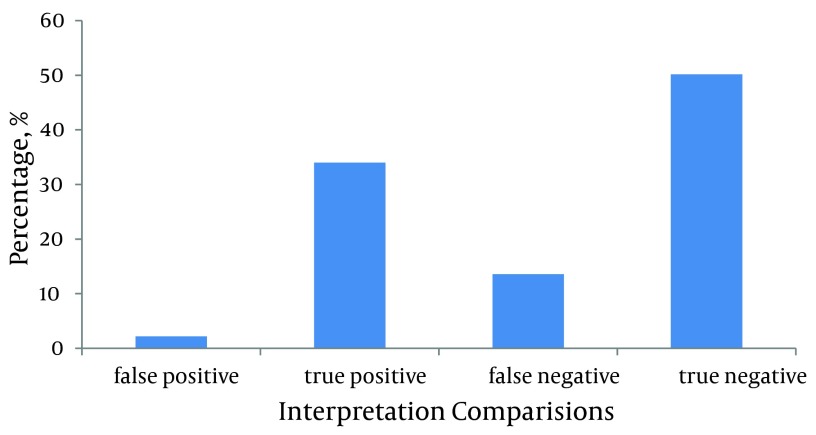
EM Residents Interpretation Correctness Percentage

On the other hand, the reports from senior residents were different from those of the radiologist, as the gold standard, for 86 (15.8%, 95%CI) cases. There were 12 (2.21%, 95%CI) false positive cases while false negative cases accounted for 74 (13.6%, 95%CI) of the discordances and the difference was statistically significant (P < 0.0001). Other statistical attributes related to interpretations from senior residents had a sensitivity of 71.4%, specificity of 95.8%, PPV of 93.9% and NPV of 78.7%. Therefore PLR and NLR were calculated to be 17 and 0.29 respectively (Figure 2).

## 5. Discussion

Several studies have examined emergency physicians’ skills in reading different radiologic studies (1-17). However, most of these studies have focused on plain X-rays rather than computed tomography scans. Our study is one of the few studies that assess brain CT scan interpretation by attending emergency physicians. Furthermore, in this study interpretation skills were probed for senior residents in emergency medicine. Discrepancy between X-ray readings of emergency physicians versus radiologists has been reported to be between 0.95% and 16.8% in different studies (11, 13). The discordance was even higher when specific studies such as chest X-rays were probed (13, 17, 18). Performing an observational research about radiologic studies requires a gold standard to be used as the reference comparison. In this study we invited a staff radiologist to participate in the project and report all the brain CT scans. It is worth mentioning that in the literature one can find reports of discrepancy as high as 13.2% between readings of the same radiologic study by two radiologists (9). Therefore, it is recommended to use a panel of radiologists to improve the quality of the research (5, 12, 18, 19). False radiologic interpretations have been described differently in different studies. For instance, while some studies only take false negatives into account (6, 8, 10), in others, both false negative and false positive cases were considered as misinterpretation (1). Therefore, reports of discrepancy vary between 14% and 33% for different studies (1, 3-5, 20-23). In our study both false negatives and false positives were deemed to be misinterpretation. The obvious reason for our choice for inclusion of false negatives was the potential harm that missing a patient with life threatening condition would cause. On the other hand, our rationale behind taking the false positives into account was the fact that these diagnoses would warrant further investigations and longer hospital stay and could subsequently inflict unnecessary financial burden on the patient and health system. It also adds to over-crowding of ER by delaying the diagnosis and discharge process. Based on the aforementioned criteria, we found a 16.2% and 15.8% discrepancy in reading brain CT scan studies by attending EPs and senior emergency medicine residents, respectively. In the review of the literature we found that higher number of abnormalities found in the studies is associated with higher interpretation discrepancies (1, 14, 18, 24). The proportion of abnormal findings in our study (47.6%) was comparable to Arendt et al. (43%) (4) and Alfaro et al. (47.6%) (2).Different researchers have also defined unfavorable consequences differently; thus the reported results differ vastly in some cases (3, 6, 14, 17-19, 25). Some have held all the false positive cases as non-significant (6, 8, 10, 11, 17), and false negatives were only considered as important discrepancies when they had caused adverse effects (8, 10, 13, 16, 26). Arendt et al. who reported 14.8% discrepancy, documented 41.1% potential unfavorable cases (1). However, only 6% of those actually ended up being undesirable (1). Rates of actual undesirable cases vary from 4% to 24% for different studies (3, 4).In our study our EPs with mean experience of 7 years in ED made a significant number of mistakes in interpreting brain CT scans. At the same time, the discrepancy between interpretations by senior residents and the radiologist was significant. While interpretations reported by EPs had higher sensitivity (86.5% versus 71.4%), residents provided higher specificity in their CT interpretations (91.8% versus 81.4%). Neither our study nor others have been able to find any correlation between the number of years of practice and the accuracy of interpretations of radiologic studies (6, 10, 14). Nonetheless, it has been shown that attending a 1-2 hour workshop can significantly increase the skills of physicians (3, 5) and this improvement can last as long as a year (18, 27, 28).Our study revealed that EPs and senior EM residents both make significant mistakes in their interpretations of CT scans of the head. It is worth mentioning that the interpretations in this study were not done in the crowded and stressful condition of the ER. We believe that conducting such study in a busy ER would possibly change the current findings for worse. This shortcoming could be better addressed by increasing didactic radiology training for residents during their clinical rotations.

Staff EPs can benefit from continuing medical education workshops in radiology to improve their skill levels in interpreting CT scan studies.
